# Experimental *Coxiella burnetii* infection in non-pregnant goats and the effect of breeding

**DOI:** 10.1186/s13567-020-00797-7

**Published:** 2020-05-29

**Authors:** Hendrik I. J. Roest, Annemieke Dinkla, Ad P. Koets, Jacob Post, Lucien van Keulen

**Affiliations:** 1 Department of Bacteriology and Epidemiology, Wageningen Bioveterinary Research, Lelystad, The Netherlands; 2 Department of Large Animal Health, Faculty of Veterinary Medicine, Utrecht University, Utrecht, The Netherlands; 3 Department of Virology, Wageningen Bioveterinary Research, Lelystad, The Netherlands; 4 Department of Infection Biology, Wageningen Bioveterinary Research, Lelystad, The Netherlands; 5 Present Address: Ministry of Agriculture, Nature and Food Quality, The Hague, The Netherlands

## Abstract

Q fever is a zoonosis caused by the intracellular bacterium *Coxiella burnetii*. In Europe, small ruminants are the main source of human Q fever. Small ruminant herds can be infectious during several lambing seasons. However, it is not clear how infection is maintained in a herd and what role non-pregnant animals play in the transmission of *C. burnetii*. We therefore inoculated nulliparous goats with *C. burnetii*, isolated from the outbreak of Q fever in the Netherlands, to gain a better understanding of the role of non-pregnant goats. Seroconversion and excretion of *C. burnetii* were monitored after inoculation. To study the effect of breeding on the excretion of *C. burnetii*, the goats were naturally bred and monitored during gestation and after lambing. Our results indicate that *C. burnetii* infection prior to breeding did not result in infection of the placenta nor did it affect the gestation length or the number of kids born. However, one of the ten does did excrete *C. burnetii* in the colostrum post-partum and the bacterium was detected in the mammary gland and associated lymph nodes at necropsy. This result indicates that non-pregnant goats might play a role in maintaining Q fever in a goat herd as persistent carriers of infection.

## Introduction

Q fever is a zoonosis caused by the intracellular bacterium *Coxiella burnetii*. The zoonotic impact of the disease has been shown in various outbreaks [1–3]. The Dutch Q fever epidemic was the biggest outbreak reported so far, with 4029 registered human cases during the years 2007–2010 and more than 40 000 people assumed to be infected following (bio-aerosol) exposure [2, 4]. Upon infection, clinical symptoms in humans vary from no symptoms at all to a flu-like, self-limiting disease, atypical pneumonia or hepatitis in the acute phase. In the chronic forms humans may suffer from a life-threatening endocarditis or chronic fatigue. Currently 439 chronic Dutch Q fever patients are registered [5].

In Europe, small ruminants are the main source of infection for human Q fever [6]. They excrete high amounts of *C. burnetii* during abortion or premature and end-term parturition of infected does. Humans become infected via direct or indirect contact with contaminated aerosolized birth material. In large goats herds, abortion rates can reach up to 80% of the infected pregnant animals although healthy kids can also be born [2, 6, 7].

It is not quite clear how Q fever persists in sheep or goat herds. Publications describe Q fever outbreaks in goat herds and excretion of *C. burnetii* during successive parturitions of the same animal [8–11]. However, these case studies do not clarify how pregnant does become reinfected. There are three possible scenarios. Firstly, placentas can be infected with *C. burnetii* that persist in the genital tract after an infected parturition as found by Alsaleh et al. [12]. Secondly their placental tissue can become reinfected from bacteria persisting elsewhere in the goat’s organs during the interpregnant period, for instance in the mammary tissue [13]. Thirdly, animals can be reinfected from a contaminated environment despite humoral and cellular immunity.

Experimental infections in pregnant goats, however, could not confirm the persistence of *C. burnetii* in mammary glands [7, 14, 15]. Moreover, excretion in the milk was found to be limited to 32 days post-partum [7]. Overall, field data and data from experimental infections are contradictory and do not explain how a *C. burnetii* infection is maintained in a herd.

Non-pregnant goats might play a role in maintaining Q fever in a herd. However, it is impossible to assess their role in a field case study as environmental infection conditions are not controlled and no diagnostic methods are known to assess the actual infection moment or the possible persistence of *C. burnetii* in live animals. An experimental infection is needed to elucidate the role of non-pregnant goats. Therefore, the goal of this study was to assess *C. burnetii* infection and (milk) excretion in non-pregnant nulliparous goats up to the outcome of the first pregnancy and start of lactation. In this experiment, successful inoculation was evaluated by the detection of serum antibodies and excretion was monitored via vaginal swabs, feces, colostrum and air samples. Goats were synchronized and bred, and after parturition, placenta’s, kids, mammary glands, and colostrum were investigated by *C. burnetii*-specific PCR. None of the inoculated goats excreted *C.* *burnetii* during parturition. One of the goats, however, excreted *C. burnetii* in the colostrum and *C.* *burnetii* DNA was detectable in the mammary gland and the associated lymph node.

## Materials and methods

### Inoculum

*Coxiella burnetii* strain X09003262-001 was used as previously described [7]. In summary, the strain is a representative of the Dutch *C. burnetii* outbreak strain, isolated from the placenta of a goat which aborted due to Q fever [17]. The strain was isolated using a Buffalo Green Monkey (BGM) cell culture. The mouse-infective dose (MID) was determined and prior to inoculation, the inoculum was adjusted to the required MID by dilution with culture medium. Cell culture passage 2 of the field isolate was used to ensure inoculation of phase 1 bacteria. In the inoculum, no phase 2 *C. burnetii* were detected with an immunofluorescence test that was set up with the serum of a goat with a high anti-phase 2 antibody titer but without phase 1 antibodies. The animal trail was conducted in accordance with the Dutch Law on Animal Experimentations (Wet op de Dierproeven, ID number 2013037c) and the European regulations on the protection of animals used for scientific purposes (EU directive 2010/63/EU).

### Animal experiment

#### Animals and inoculation

Twenty-four healthy, serologically Q fever negative, Alpine goats were purchased from INRAE (Institut national de recherche pour l’agriculture, l’alimentation et l’environnement, Domaine de Galle), France. Upon arrival the non-pregnant nulliparous goats were 15 weeks old and tested serological negative for antibodies against *C.* *burnetii* (LSIVET RUMINANT milk/serum Q-fever ELISA kit, LSI, Lyon, France) and *Chlamydia abortus* (Chekit Chlamydophila abortus antibody test kit, IDEXX laboratories B.V., Hoofddorp, the Netherlands). After 1 week of acclimatization, 16 goats were divided over two animal rooms in the animal biosafety level 3 (aBSL3) facility. Goats were intranasally inoculated with 1 mL containing 10^6^ MID *C. burnetii* with a nozzle in the left nostril with the right nostril closed during forced inhalation. Eight negative control animals remained outside the aBSL3 facilities and were intranasally inoculated with 1 mL of culture medium. This inoculation procedure was used previously to inoculate pregnant goats. This resulted in a successful infection with *C. burnetii* resulting in pathology, excretion of *C. burnetii* and abortion [7].

At 49 days post-inoculation (dpi), after repeated negative testing of individual blood and fecal samples and air samples for *C. burnetii* DNA, all *C. burnetii* inoculated animals were moved from aBSL3 to two animal rooms in the aBSL2 facility for estrus synchronization and breeding. The eight control animals remained in their initial aBSL2 animal room and underwent the same synchronization and breeding procedures as the *C. burnetii* inoculated animals (Table 1).

**Table 1:** **Overview of the experimental set up and results of the*Coxiella burnetii*infection in goats from the start of the experiment till pregnancy confirmation**

Status		Inoculated animals												Negative controls			
Goat ID:		7917	7919	7920	7922	7923	7924	7925	7926	7928	7929	7931	7932	8321	8322	8323	8324
Day in study	Week in study																
−7	−1	Arrival in aBSL2; feces sample: *C. burnetii* DNA neg; serum sample: see Figure 1												Arrival in aBSL2			
0	0	Move to aBSL3; inoculation *C. burnetii*; feces sample: *C. burnetii* DNA neg; serum sample: see Figure 1															
7	1	For all animals EDTA blood sample, feces sample, air sample per room:*C. burnetii* DNA neg; serum sample: see Figure 1															
14	2	For all animals EDTA blood sample, feces sample, air sample per room: *C. burnetii* DNA neg; serum sample: see Figure 1															
21	3	For all animals EDTA blood sample, feces sample, air sample per room: *C. burnetii* DNA neg; serum sample: see Figure 1															
28	4	For all animals EDTA blood sample, feces sample, air sample per room: *C. burnetii* DNA neg; serum sample: see Figure 1															
35	5	For all animals EDTA blood sample, feces sample, air sample per room: *C. burnetii* DNA neg; serum sample: see Figure 1															
42	6	For all animals EDTA blood sample, feces sample, air sample per room: *C. burnetii* DNA neg; serum sample: see Figure 1															
49	7	For all animals EDTA blood sample, feces sample, air sample per room: *C. burnetii* DNA neg; serum sample: see Figure 1 → move to aBSL2															
91	13	11.5 h light															
98	14	11 h light												11.5 h light			
105	15	10.5 h light												11 h light; intravaginal progesterone sponge administered on D 108			
112	16	10 h light; intravaginal progesterone sponge administered on D 115												10.5 h light; 0.2 mL Estrumate i.m. and 1.25 mL Folligonan i.m. on D 117			
119	17	10 h light; 0.2 mL Estrumate i.m. and 1.25 mL Folligonan i.m. on D 124												10 h light; progesterone sponge removed on D 119 / breeding on D 120			
126	18	Progesterone sponge removed on D 126 / breeding on D 127															
133	19																
140	20	D 141: air sample per room: *C. burnetii* DNA neg; for all animals: vaginal swab: *C. burnetii* DNA neg															
147	21											Breeding					
154	22	D 155: air sample per room: *C. burnetii* DNA neg; for all animals: vaginal swab: *C. burnetii* DNA neg															
161	23	Pregnancy check echoscopy												Pregnancy check by echoscopy			

ID: identification, aBSL: animal biosafety level, neg: negative, h: hour, i.m.: intramuscular, D: day in study.

Goats had ad libitum access to water and hay and were fed limited amounts of concentrate on a once daily basis. The animals were group housed in aBSL2 and aBSL3 compartments with regulated temperature and humidity, with a 12/12 h light/dark cycle unless stated differently, in accordance with EU directive 2010/63/EU.

#### Estrus synchronization and breeding

From 91 dpi onwards, light was restricted to 11.5 hour (h) per day and reduced every week with half an hour till 10 h a day at 112 dpi (Table 1) to mimic the shortening of day length during the change of season. At 115 dpi, a Progesterone vaginal sponge (Chronogest CR, MSD-Animal Health, Boxmeer, the Netherlands) was inserted and at 124 dpi 0.2 mL Estrumate (MSD-Animal Health) and 1.25 mL Folligonan (MSD-Animal Health) were injected intramuscularly. On 126 dpi the sponge was removed and on 127 dpi all goats were naturally mated by a serologically Q fever negative tested buck that had not mated before. One goat was mated again 3 weeks later when she came into estrus again. On 161 and 168 dpi, gestation was checked by ultrasound scanning. Twelve *C. burnetii* infected goats and 4 control animals that were positively scanned for gestation were then moved to two boxes in aBSL3 facilities where they stayed until parturition (6 *C. burnetii* infected mixed with 2 control animals in each box, Table 2). In this step the control animals became sentinel animals. General health was monitored by daily clinical inspection of behavior, appetite, and consistency of the feces.

**Table 2:** **Overview of the experimental set up and results of the*Coxiella burnetii*infection in goats during pregnancy**

Status		Inoculated animals												Negative controls/sentinel			
Goat ID:		7917	7919	7920	7922	7923	7924	7925	7926	7928	7929	7931	7932	8321	8322	8323	8324
Day in study	Week in study																
168	24	Pregnancy check by echoscopy, move to aBSL3, 2 rooms with both inoculated and negative control goats (sentinel animals)															
196	28	Sentinel animals: no seroconversion															
231	33	Sentinel animals: no seroconversion															
259	37	Sentinel animals: no seroconversion															
266	38	Air sample per room: *C. burnetii* DNA neg, vaginal swab and feces sample: *C. burnetii* DNA neg; sentinel animals: no seroconversion															
273	39	Air sample per room: *C. burnetii* DNA neg, vaginal swab and feces sample: *C. burnetii* DNA neg; sentinel animals: no seroconversion															
280	40	Air sample per room: *C. burnetii* DNA neg, vaginal swab and feces sample: *C. burnetii* DNA neg; sentinel animals: no seroconversion															

ID: identification, aBSL: animal biosafety level, neg: negative.

### Sampling

At −7 dpi till 49 dpi jugular blood and rectal feces were sampled weekly from each *C. burnetii* inoculated goat. The air was sampled using an air sampler (MD 8 airscan Air Sampler Sartorius, Goettingen, Germany), as described previously [7]. After breeding and co-housing of infected and control animals, weekly blood samples were taken from the control animals to check for seroconversion (LSIVET RUMINANT milk/serum Q-fever ELISA kit, LSI). In addition, feces samples, vaginal swabs and air samples were taken around the expected parturition date (between 141 and 155 days of gestation, D268 and D282 of the experiment). Just after parturition, colostrum samples were taken from each goat from both teats.

### Necropsy

Shortly after parturition, both does and kids were euthanized by intravenous injection of 50 mg/kg sodium pentobarbital (Euthasol^®^, ASTfarma, Oudewater, the Netherlands) and subsequent exsanguination. At necropsy, the following tissues were sampled from the does: palatine tonsil, retro-pharyngeal lymph node, kidney, liver, spleen, lung, bronchial and mediastinal lymph nodes, mammary gland and the draining inguinal lymph node, iliacal lymph nodes, bone marrow, both ovaries, both oviducts, 3 caruncles from each uterus horn, non-caruncular mucosa from each uterus horn and colostrum. The following were sampled from each kid and afterbirth: spleen, liver, kidney, lung, umbilical cord, 3 cotyledons, 3 areas of non-cotyledonary allantochorion and blood.

### Serology

In serum samples taken at −7 dpi till 49 dpi *C. burnetii* phase 1 and phase 2 IgM- and IgG-specific antibodies were detected in an ELISA format as described earlier [16]. In summary, *C.* *burnetii* phase 1 and phase 2 ELISA-specific plates were purchased from Virion/Serion (Serion ELISA classic *Coxiella burnetii* phase 1 and phase 2, Würzburg, Germany). Plates were incubated with 100 µL 1:160 diluted serum in phosphate buffered saline (PBS), pH 7.2 with 0.5 mL 10% (v/v) tween 80 (PBS-Tw) for 1 h at 37 °C. After incubation, plates were washed automatically (Schleicher, Dassel, Germany), 6 times with 1400 µL of 0.5‰ Tween 20 in water and incubated for 1 h at 37 °C with 100 µL of diluted alkaline phosphatase-conjugated antibodies. For the detection of IgM antibodies, rabbit anti-goat IgM antibodies (Bioconnect, Huissen, the Netherlands) were used, 1:1000 diluted in PBS-Tw and 0.5 M NaCl for the detection of phase 1 antibodies or 1:5000 diluted for the detection of phase 2 antibodies. For the detection of IgG antibodies rabbit F(abʹ)2 anti-goat IgG (H/L) (Bioconnect) were used, 1:2000 diluted for the detection of phase 1 antibodies or 1:4000 diluted for the detection of phase 2 antibodies. After incubation with the conjugate, plates were washed as described above, 100 µL of para-nitrophenylphosphate substrate (Virion/Serion) per well was added, and the reaction was stopped after 30 min at 37 °C with 100 µL of 1.2 N sodium hydroxide (Virion/Serion). The optical density (OD) was measured at 405 nm (EL 808 Ultra microplate reader, Bio-tek Instruments, Winooski, USA). On each plate the same negative and positive control serum was tested in duplicate per phase/Ig combination. Results of the serum were given related to the average positive control OD corrected for the average negative control OD. The average *C. burnetii*-specific antibody responses upon *C. burnetii* inoculation of 16 non-pregnant nulliparous goats were compared to the antibody response on 0 dpi.

### PCR for detection of *Coxiella burnetii*

DNA was extracted from tissues (20 mg), feces (20 mg), vaginal mucus swabs (swab tip), EDTA blood (200 μL), colostrum (200 μL), and environmental samples (filter part the size of a swab tip) using a DNA tissue kit (DNeasy Blood & Tissue Kit, Qiagen, Venlo, the Netherlands) according to the manufacturer’s instructions, as previously described [7]. All samples were subjected to a quantitative PCR (qPCR) targeting a single copy gene encoding a *C.* *burnetii*-specific hypothetical protein (gene bank number AY502846) using the forward primer 5′-ATAGCGCCAATCGAAATGGT-3′, the reverse primer 5′-CTTGAATACCCATCCCGAAGTC-3′, and the NED-labelled probe 5′-CCCAGTAGGGCAGAAGACGTTCCCC-3′. An inhibition control (IC) was constructed using primers for the IS1111a element and a dedicated VIC-labelled probe, as previously published [17]. PCR was performed on a 7500 Fast Real Time PCR system (Applied Biosystems, Waltham, USA), using 400 nmol/L of primers and 200 nmol/L of probes in 7 μL PerfeCTa Multiplex qPCR Super mix, UNG (2X) with Low Rox dye (Quanta Biosciences, Gaithersburg, USA), 1 μL of IC, 5 μL of sample and 7 μL of water. An initial UDG incubation for 5 min at 45 °C and denaturation/activation for 60 s at 95 °C was followed by 50 cycles of denaturation for 10 s at 95 °C and annealing for 30 s at 60 °C. A Ct value of > 40 was scored as negative and a Ct value < 40 scored as positive.

### Statistical analysis

For the serology data, differences between dpi were analyzed using one-way ANOVA. A *p*-value < 0.01 (**) was considered statistically significant compared to 0 dpi. The maximum 95% probability of abortion in the ten inoculated goats was calculated as 0.238.

## Results

### *Coxiella burnetii* infection and excretion

**Figure 1:**
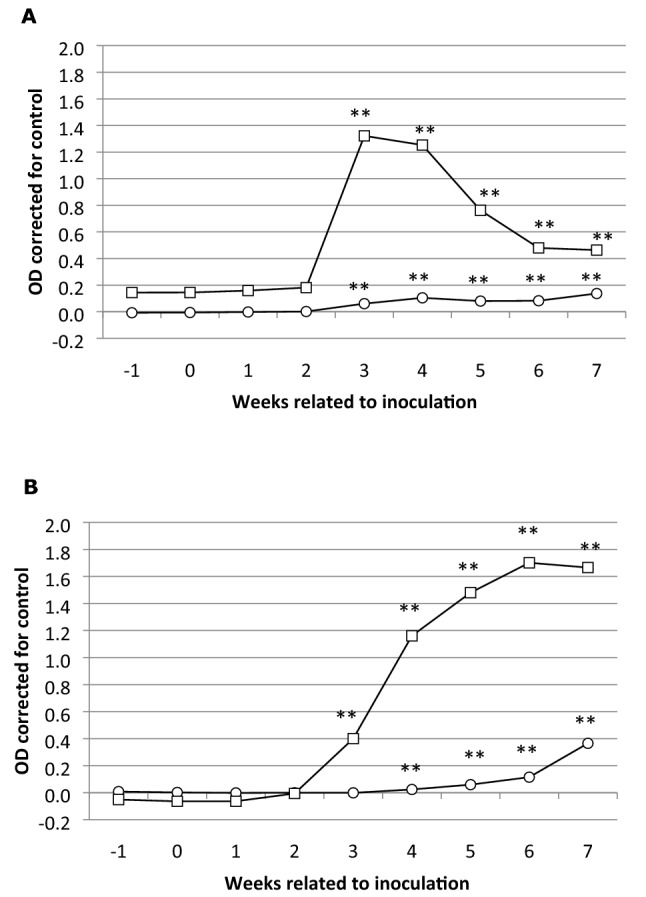
**Average*Coxiella burnetii*-specific antibody response upon*C. burnetii*inoculation of 16 non-pregnant nulliparous goats.A** IgM phase 1 (open circle) and phase 2 (open square) response. **B** IgG phase 1 (open circle) and phase 2 (open square) response.

To study the role of non-pregnant goats in Q fever herd dynamics, we inoculated nulliparous goats with *C. burnetii* and assessed the *C. burnetii* infection by their IgG and IgM antibody response. All *C. burnetii* inoculated goats showed an antibody response indicating a successful infection with *C. burnetii* (Figure  1). The control (sentinel) animals remained seronegative throughout the experiment (Table 2).

PCR analysis of blood samples showed that none of the infected goats developed a bacteremia during the first 7 weeks after inoculation and none of the goats excreted *C.* *burnetii* DNA in their feces up to 7 weeks post inoculation. In the air of the animal rooms, *C. burnetii* DNA was not detected up to 7 weeks post inoculation (Table 1).

After breeding vaginal swabs were negative for *C. burnetii* DNA at 141 and 155 dpi (14 and 28 days after breeding). Air samples from the two BSL3 boxes were also negative for *C. burnetii* DNA, so no excretion of *C. burnetii* was detected (Table 1). During pregnancy air samples, vaginal swabs and feces samples remained negative for *C. burnetii* DNA (Table 2).

### Pregnancy outcome and *Coxiella burnetii* excretion

Two infected goats appeared to be non-pregnant at the time of parturition (most likely due to pseudo-pregnancy at the time of echoscopy). One infected goat was still pregnant at termination of the experiment due to non-conception at first mate. The remaining nine infected goats delivered healthy lambs after an average gestation of 152.89 days (95%CI 151.49–154.29) with on average 2.00 kids (95%CI 1.49–2.51) and the four control goats kidded after an average gestation of 153.50 days (95%CI 152.23–154.77) with on average 1.75 kids (95%CI 0.81–2.69, Table 3). So all pregnant goats kidded full term and no significant differences in gestation length or number of kids was measured between *C. burnetii* inoculated goats and control goats. The placentas of the inoculated does gave no macroscopic indication for inflammation and *C. burnetii* DNA was not detected in the cotyledon or inter-cotyledonary allantochorion. The sentinel goats also showed no macroscopic lesions of the placenta and DNA of *C. burnetii* was not detected in the placentas. However, in one inoculated goat, *C. burnetii* DNA was detected in the colostrum (Ct value: 34.05) and the mammary gland (Ct value: 30.26). Due to this result, the organs of this doe and her kids were investigated using *C. burnetii* PCR. None of the other tissues were positive except for the samples from the mammary gland (Ct value: 28.94), inguinal lymph node (Ct value: 39.14), and colostrum (Ct value: 32.50) indicating that *C. burnetii* was present in the mammary tissue of this goat (Table 3). So, although *C. burnetii* DNA could not be detected in the placenta after parturition, we were able to detect *C. burnetii* DNA in the colostrum and mammary glands of one out of 10 inoculated goats after parturition. No *C. burnetii* was detected in the colostrum or mammary gland and lymph nodes of the sentinel animals (Table 3).

**Table 3:** **Results of the pregnancy outcome of the*Coxiella burnetii*infection in goats and of the negative controls/sentinel animals**

Status	Inoculated animals												Negative controls			
Goat ID:	7917	7919	7920	7922	7923	7924	7925	7926	7928	7929	7931	7932	8321	8322	8323	8324
Pregnancy outcome	154d 3kl^a^	Not preg	151d 2kl	152d 2kl	153d 2kl	157d 1kl	154d 2kl 1kd^b^	150d 2kl 1kd^c^	154d 1kl	Not preg	nd 1k^d^	151d 2kl	154d 1k^e^	152d 2kl	155d 3kl	153d 1kl
Average pregnancy outcome	Gestation: 152.89d (95%CI 151.49–154.29); 2.00 kids (95%CI 1.49–2.51)												Gestation: 153.50d (95%CI 152.23–154.77); 1.75 kids (95%CI 0.81–2.69)			
Milk sample	neg	neg	neg	neg	neg	neg	neg	neg	neg	ns	ns	pos	neg	neg	neg	neg
Mammary gland	neg	ns	neg	neg	neg	neg	neg	neg	neg	neg	neg	pos	neg	neg	neg	neg
Inter-cotyledonal placenta k1	neg	ns	neg	ns	neg	neg	neg	neg	neg	ns	neg	neg	neg	neg	neg	neg
Cotyledonal placenta k1	neg	ns	neg	ns	neg	neg	neg	neg	neg	ns	neg	neg	neg	neg	neg	neg
Inter-cotyledonal placenta k2	neg			ns			neg					neg	neg	neg	neg	
Cotyledonal placenta k2	neg			ns			neg					neg	neg	neg	neg	
Mammary gland												pos				
lnn mammary gland												pos				
Milk												pos				

ID: identification, neg: negative, pos: positive, d: days of pregnancy, CI: confidence interval, not preg: not pregnant, nd: no delivery, kl: kid(s) alive, kd: kid(s) dead, k: kid, k1: kid 1, k2: kid 2, ns: no sample.

^a^1 week kid euthanised; ^b^choked in placenta; ^c^durable partus; ^d^in utero; ^e^mummified kid (10 cm).

## Discussion

The goal of this study was to investigate *C. burnetii* infection and excretion in non-pregnant nulliparous goats up to the outcome of the first pregnancy and colostrum production in order to assess the role of non-pregnant goats in herd Q fever dynamics. Although several inoculation studies on pregnant goats have been published [7, 14–16, 18], no studies are available on non-pregnant goats inoculated under experimental conditions. As the inoculation with 10^6^ MID *C. burnetii* in pregnant goats resulted in pathology, excretion of *C. burnetii*, abortion and seroconversion [7, 16], we assessed this procedure as successful and one of the representatives of the field situation, for that reason we used it also to evaluate the goal in this study. Our results indicate that inoculation of non-pregnant goats resulted in an antibody response comparable to the response in inoculated pregnant goats, indicating infection. This *C. burnetii*-specific antibody response showed a significant increase in phase 2 IgM and IgG after 14 dpi. This increase is comparable to the phase 2 IgM and IgG antibody increase in pregnant goats after inoculation with 10^6^ MID *C. burnetii* [16]. The IgM phase 1 antibody response significantly increased after 21 dpi, whereas the IgG phase 1 showed a significant increase after 28 dpi. The phase 1 IgG response is also comparable with the previous study, although the phase 1 IgM response was slightly lower in that data [16].

After intranasal inoculation and during breeding, no *C. burnetii* DNA was detected in feces or environmental samples. This indicates that *C. burnetii* was not excreted after infection and during estrus. Although shedding in non-pregnant goats was reported in several field studies, this was always related to previous kidding with excretion of *C.* *burnetii* [9, 19, 20]. Experimental studies show that *C. burnetii* was not excreted before parturition [7], which is in line with the results in this study. Since *C. burnetii* was not detected by PCR in any of the fecal and air samples, the goats could safely be moved from BSL3 facilities to BSL2 facilities, as the risk for spreading *C. burnetii* in the environment was negligible. This is also important for hobby goat owners and goat farmers as, although goats may be kept in a contaminated environment, non-pregnant goats pose no risk for their environment and as such pose no risk for public health and veterinary health, provided that these animals are not bred.

Breeding and gestation of non-pregnant *C. burnetii* infected goats resulted in a term delivery of a normal number of healthy kids without the excretion of *C. burnetii* DNA or infection of the placenta/afterbirth. In addition, non-infected pregnant sentinel goats, co-housed with the inoculated goats, did not show seroconversion, fecal excretion of *C. burnetii* or abortion due to possible shedding of *C burnetii* by the infected goats. Given the preference of *C.* *burnetii* for trophoblast cells, as demonstrated in earlier studies [7, 14], one could have expected that as soon as trophoblast cells arise during the first pregnancy then these cells could have been infected from a source of persistent infection in the doe. The fact that no abortion or infection of placental tissues occurred could have two reasons. Firstly, *C. burnetii* was not persisting in the goat’s tissues. As shown above, non-pregnant goats develop an immune response upon infection and this immune response may well be effective in eliminating the infection. However, this does not seem to be the case for one out of 10 goats, in which *C. burnetii* DNA was detected in the mammary gland, associated lymph nodes and colostrum. A second explanation could be that in persistently infected goats, circulation of bacteria does not occur or bacteria are eliminated when entering the circulation and so trophoblast cells are not infected via this route. This is supported by the data that revealed no indications for circulating *C. burnetii* immediately after infection, although the bacteremia might remain under the detection limit of the sampling and test. As the trophoblasts were not infected, *C. burnetii* is not excreted upon delivery and therefore the risk non-pregnant goats pose in a herd can be assessed as low.

Although the number of goats in this study is relatively small, which hamper the negative predictive value, we anticipate that infection of non-pregnant goats does not result in substantial excretion of *C. burnetii* upon breeding. In earlier experiments with pregnant goats, the success rate of inoculation and subsequent *C. burnetii* excretion upon delivery was 100% [7, 14, 15]. We therefore assumed that breeding is the ultimate test for the infection status of goats. This, however, appeared not to be true. Even if goats do not excrete *C. burnetii* via the placenta and birth fluids, goats can still be infected and excrete *C. burnetii* via the colostrum.

In one of the ten inoculated goats, *C. burnetii* DNA was detected in the mammary gland, associated lymph nodes and colostrum. This is remarkable as *C. burnetii* was not excreted with the placenta or kids upon delivery. The result is, however, in line with earlier studies, which show excretion of *C. burnetii* DNA in the milk and persistence of DNA of the bacterium in the mammary gland [13]. These earlier studies, however, provided no clues about the origin of the *C. burnetii* persisting in the mammary gland: did these enter during an infected pregnancy or were they already present before the pregnancy? Our results indicate that the latter is possible. The only origin of the *C.* *burnetii* in our study is the inoculation before breeding, so in non-pregnant goats. The preference of *C.* *burnetii* for mammary tissues was already demonstrated in an in vitro comparison study in which the susceptibility of different epithelial cells for *C. burnetii* was assessed [21]. The persistence of DNA and presumably infectious *C.* *burnetii* in the mammary gland, and especially the excretion in the colostrum, potentially results in infection of the suckling kids and contamination of the environment. From this contaminated environment, highly susceptible pregnant goats can be infected for example via automatic milking systems. In this way non-pregnant goats that are infected with *C. burnetii* before breeding, could play a role in the dynamics of Q fever in a goat herd although the exact attribution of pre-bred infected goats cannot be derived from this study. This risk can be further reduced by vaccinating the kids as early as possible, which Muleme et al. have already suggested as an approach for eradicating Q fever from an infected herd [22].

## Data Availability

The datasets during and/or analysed during the current study are available from the corresponding author on reasonable request.

## Abbreviations

MID: mouse-infective dose
aBSL: animal biosafety level
dpi: days post-inoculation
h: hour
IC: inhibition control
OD: optical density
ID: identification
neg: negative
pos: positive
i.m.: intramuscular
D: day in study
d: days of pregnancy
CI: confidence interval
nd: no delivery
not preg: not pregnant
kl: kid(s) alive
kd: kid(s) dead
k: kid
k1: kid 1
k2: kid 2
ns: no sample
